# Out of Lust or Jealousy: The Effects of Mate-Related Motives on Study-Time Allocation to Faces Varying in Attractiveness

**DOI:** 10.1371/journal.pone.0132207

**Published:** 2015-06-29

**Authors:** Weijian Li, Yuchi Zhang, Fengying Li, Xinyu Li, Ping Li, Xiaoyu Jia, Haide Chen, Haojie Ji

**Affiliations:** 1 Institute of Psychology, Zhejiang Normal University, Jinhua, Zhejiang Province, P.R. China; 2 Department of Psychology and Behavioral Science, Zhejiang University, Hangzhou, P.R. China; 3 Institute of Developmental Psychology, Beijing Normal University, Beijing, P.R. China; Macquarie University, AUSTRALIA

## Abstract

Although a growing number of empirical studies have revealed that activating mate-related motives might exert a specific set of consequences for human cognition and behaviors, such as attention and memory, little is known about whether mate-related motives affect self-regulated learning. The present study examined the effects of mate-related motives (mate-search and mate-guarding) on study-time allocation to faces varying in attractiveness. In two experiments, participants in mate-related priming conditions (Experiment 1: mate-search; Experiment 2: mate-guarding) or control conditions studied 20 female faces (10 highly attractive, 10 less attractive) during a self-paced study task, and then were given a yes/no face recognition task. The finding of Experiment 1 showed that activating a mate-search motive led the male participants to allocate more time to highly attractive female faces (i.e., perceived potential mates) than to less attractive ones. In Experiment 2, female participants in the mate-guarding priming condition spent more time studying highly attractive female faces (i.e., perceived potential rivals) than less attractive ones, compared to participants in the control condition. These findings illustrate the highly specific consequences of mate-related motives on study-time allocation, and highlight the value of exploring human cognition and motivation within evolutionary and self-regulated learning frameworks.

## Introduction

An evolutionary perspective suggests that people possess fundamental social motives (e.g. self-protection, mate acquisition, mate retention, and child rearing) shaped by natural selection to produce behaviors that increase reproductive fitness [1–3]. A growing number of empirical studies revealed that if these motivational states were aroused, they would exert a specific set of consequences for human cognition and behavior in relation to specific kinds of fitness-relevant stimuli in the social environment [1]. Mate-related motives, as one of fundamental social motives, might also be expected to have functionally specific effects on downstream social behaviors [4–10]. For instance, Maner et al. adopted a visual cuing method to assess participants’ attentional adhesion to potential mates or rivals when their mate-related motive was activated [4]. In their Experiment 1 and 2, activating a mate-search motive increased the time to shifting a participant’s attention away from attractive members of the opposite sex (i.e., desirable mating partners). Experiment 3 extended the previous studies by examining processes associated with different mating-related motive: mate-guarding goal (i.e., guarding against reproductive threats posed by same-sex rivals). This experiment revealed that mate-guarding priming increased attentional adhesion to physically attractive members of one’s own sex. These experiments suggested that mate-related motives priming might guide basic, lower order social perception (i.e., human attention) [4]. Subsequent research replicated these findings in different social contexts. Researchers also found that mate-related motives priming influenced not only basic-level cognitive processes, such as the way to categorize the opposite sex’s character by facial attractiveness [6] or memory of attractive opposite-sex faces [9], but also higher-level cognition and behavior, such as conspicuous consumption tendencies [5] and creative activities [10].

Despite this growing literature, little is known about how mate-related motives interact with goal-relevant stimuli to influence self-regulated learning (SRL). This is the focus of the current study. SRL is learning that is guided by metacognition (thinking about thinking), strategic action (planning, monitoring, and evaluating personal progress against a standard), and motivation to learn, which plays a crucial role in the complex social life of humans [11–18]. Our ancestors and current humans both have been living in a world flooded with large amounts of information (e.g., the location of a predator or prey’s lair, remembering the name or face of the pretty girl at a party) [19]. Due to the limited cognitive and temporal resources, individuals had to allocate precious resources to remember valuable information. Ineffective ways to learn fitness-relevant information could have serious or even fatal consequences for an individual’s life. Thus, it is important for us to optimize our time and effort in a study situation [20, 21].

There are several features of SRL. Firstly, SRL is goal-oriented, that is, “directly toward reducing a discrepancy between current perceived state and a goal relevant to performance or learning” [12]. Secondly, individuals regulate their learning behaviors flexibly based on the different study situations or motivational states [22–24]. Previous research found that both young and old participants allocated a greater amount of study time to high-value words than to low-value words (i.e., each word was displayed with a point value ranging from 1 to 30 points) and thus maximized the likelihood of achieving their learning goals [24]. Finally, although much of individuals’ SRL involves self-awareness and reflection, self-regulation itself does not require that regulators are conscious of their ongoing efforts [12]. In one of our previous studies, we found that individuals preferred to first study large-font items rather than small-font items, and this font-size effect occurred without awareness [25].

Study-time allocation is one of the most frequently used methods to measure the SRL process [20–24]. One of the well-established paradigms is to record how much time individuals spend on a given learning item (i.e., self-paced study time), and the differences in study time allocated to various types of learning material would reflect individuals’ control of their study process. A highly attractive female face implies a high reproductive value, which represents an ideal mate for a male, or a main intrasexual rival for other females [1, 4, 26–27]. Thus, face attractiveness is a cue of health and fecundity [28, 29]. Functionally speaking, remembering highly attractive female faces is vital for males and females in mating-related situations (e.g., mate-search and mate-guarding) [1]. From the perspective of study-time allocation, allocating more time to highly attractive faces than to less attractive ones could enhance an individual’s likelihood of successfully remembering those attractive faces, eventually helping the individual to obtain his or her mating goals (finding an ideal mate or guarding their mate from other rivals) [1, 22]. Thus, study-time allocation could have a relevant functional role in human reproductive fitness. Metcalfe and Jacobs used this analogy to argue that people try to optimize their study-time allocation in a learning environment, similar to other species that try to optimize their efficiency in foraging situations. Humans’ success as learners depends on the effectiveness of these strategies, just as animals’ evolutionary fitness depends on their foraging effectiveness [20]. Since the mate-related motives induce functional processes, and since spending more time on highly attractive female faces in a mate-related environment could be beneficial for individuals to solve their mating problems, it is natural to predict that mate-related motives affect study-time allocation to female faces varying in attractiveness.

In the present study, we used well-validated priming procedures to activate particular mating-related motives and investigated whether activating the mate-search motives (Experiment 1) would have a specific influence on study-time allocation to female faces varying in attractiveness. To reproduce successfully (i.e., to produce viable offspring and raise them to reproductive age) [1], individuals not only need to find an ideal mate but also need to guard their mates from same-sex rivals [4]. Adopting the same face material and study procedure, Experiment 2 (mate-guarding) was designed to conceptually extend the findings of Experiment 1 by investigating the study-time allocation effects on female participants. We predicted that participants whose mate-related motives were activated would allocate more time to studying highly attractive female faces.

## Experiment 1

**Fig 1:**
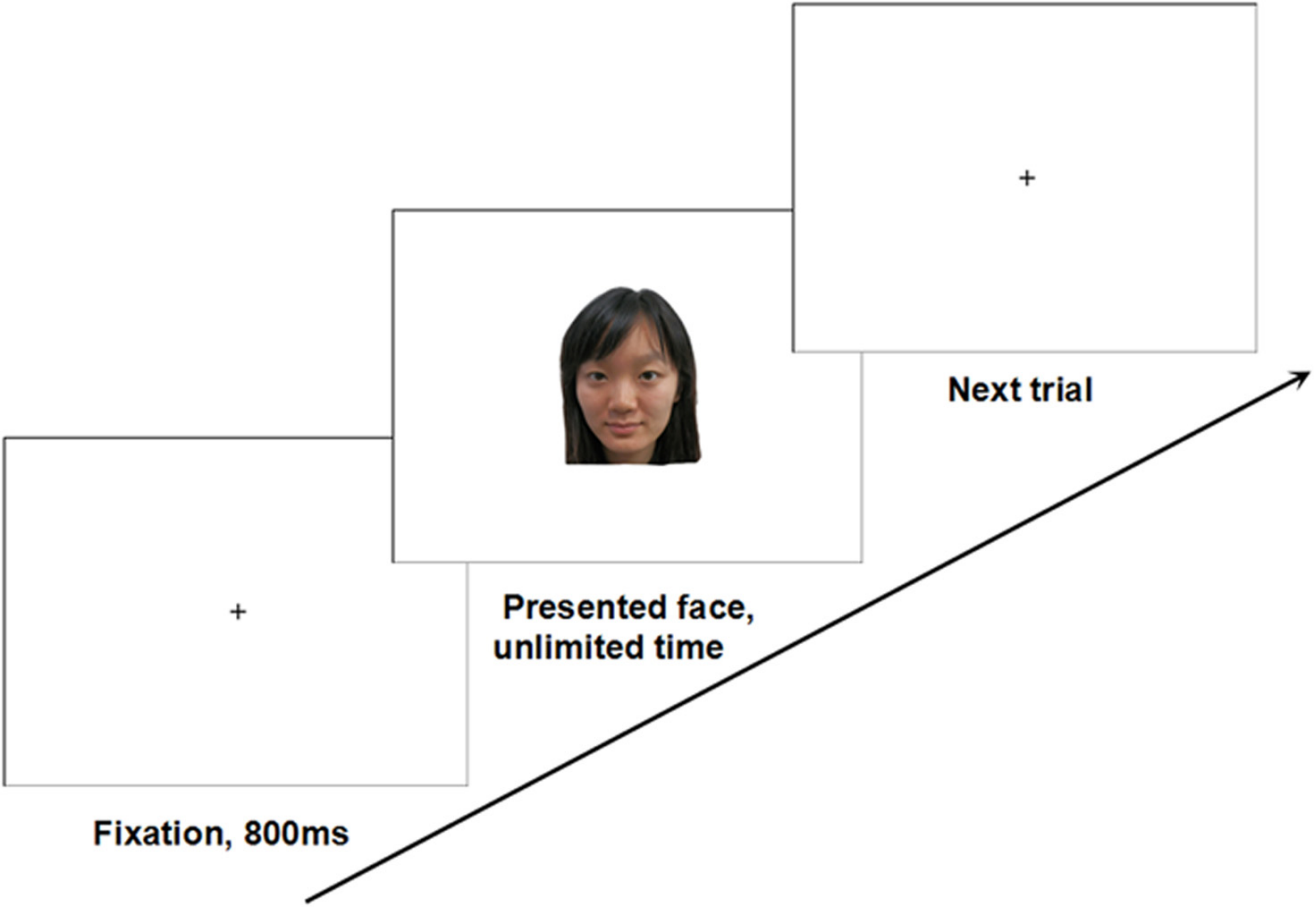
Flow diagram of Experiment 1. (due to the copyrights of the face database, the face image is a sample provided by a volunteer, and not the actual experimental material).

**Fig 2:**
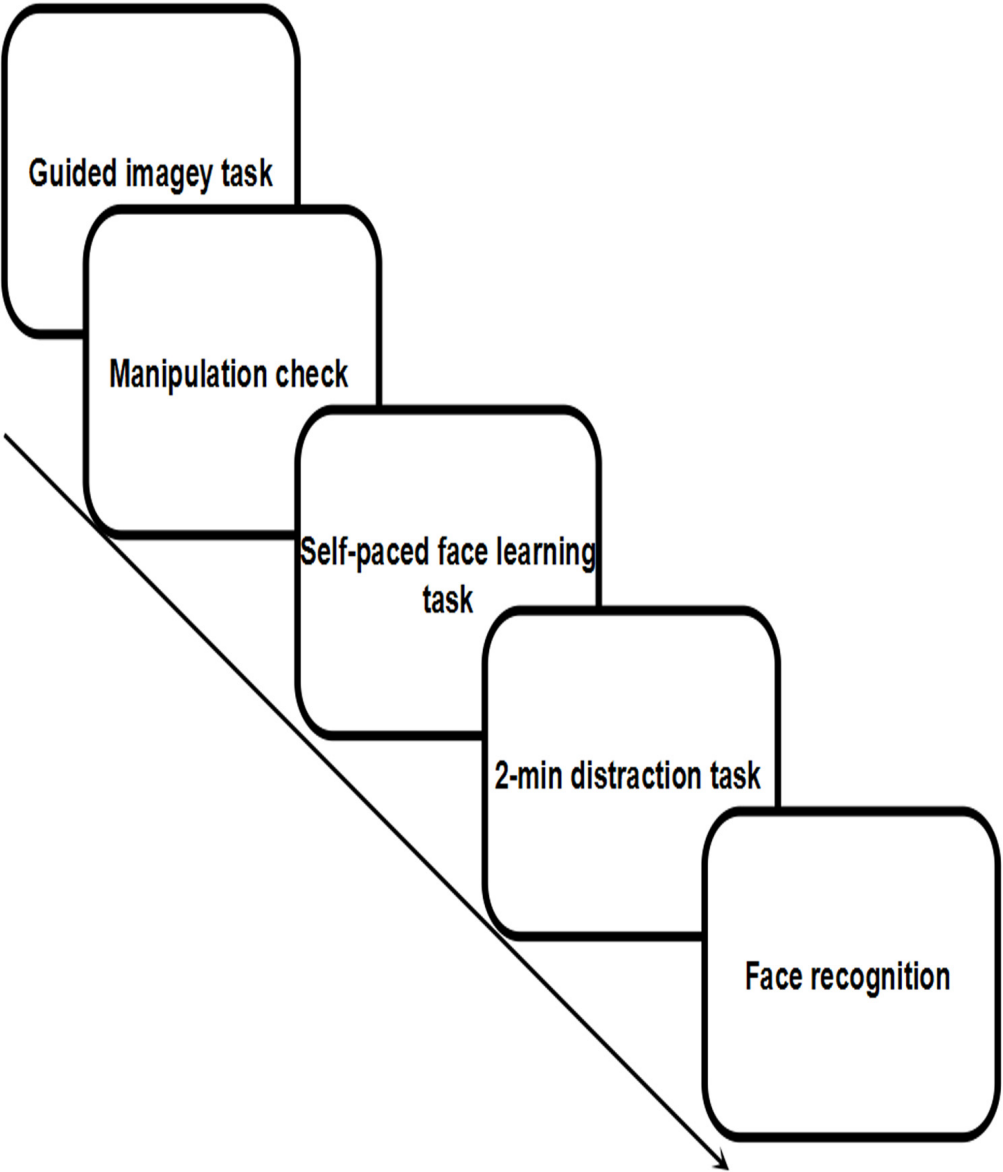
Flow diagram of self-paced study task.

In this experiment, we manipulated the priming condition (mate-search vs. happiness-control) using a guided imagery procedure [4, 6]. After the priming procedure, male participants performed a revised version of the self-paced face-learning task [30], which provided a measure of study-time allocation. Figs 1 and 2 show the flow diagram of Experiment 1 and the self-paced face-learning task (Figs 1 and 2). We hypothesized that activation of a mate-search motive would lead male participants to allocate more time to studying highly attractive female faces.

### 2.1. Methods

#### 2.1.1. Participants

This study was approved by Zhejiang Normal University Research Ethics Review Committee.The individual in this manuscript has given written informed consent (as outlined in PLOS consent form) to publish these case details. Thirty male Chinese undergraduate students (mean age = 19.59 years, *SD* = 1.37 years) were recruited and provided written consent before the experiment. All participants were right-handed single males with normal vision. They were randomly assigned to either the mate-search or happiness-control condition and tested individually by the same male experimenter. Upon completion of the experiment, they received financial compensation. During the experiment, one participant in the happiness-control group quit the experiment for personal reasons.

#### 2.1.2. Materials

Forty color photographs of Chinese female faces that displayed neutral expressions were used. All face photographs were frontal views, which were taken from the Oriental Face Database (OFD) [31]. We standardized each image in size (320 x 307 pixels) and removed the background of the photograph using Adobe Photoshop CS5. In a pilot study, 31 independent participants (15 female, 16 male, mean age = 20.2 years) were asked to rate the attractiveness of each female face on a 7-point Likert scale (1 = unattractive, 7 = highly attractive). We then divided all photographs into two sets (i.e., highly attractive and less attractive faces). An independent-samples *t*-test confirmed a significant difference in attractiveness between the highly attractive (*M* = 4.52, *SE* = 0.14) and less attractive face set (*M* = 2.23, *SE* = 0.07), *p* < 0.001.

#### 2.1.3. Design and procedure

A 2 (attractiveness: highly attractive vs. less attractive) × 2 (condition: mate-search vs. happiness-control) mixed design was adopted, with condition as the between-subjects factor and attractiveness as the within-subject factor.

After signing their consent forms, participants were told that the study investigated imagination and memory. Participants were randomly assigned to either the mate-search or happiness-control condition. Participants in the mate-search condition were requested to imagine that their student society would be arranging a dating party in a few days and they were invited together with some single female students. Participants in the happiness-control condition were requested to imagine that they were arranging a trip with their families for the next vacation. All participants were asked to visualize each event based on the script. After finishing the guided imagery procedure, all participants were asked to report the valence and arousal level of their emotions, romantic feelings, mating motivation, and sexual arousal on a 7-point Likert manipulation check scale [32].

Next, participants in the two conditions were asked to study 20 female faces with different instructions. For the mate-search condition, participants were told that the face images belonged to the single female students who would join the imaginary dating party. For the happiness-control condition, participants were told that the face images belonged to strangers whom they would meet during the imaginary family trip. All participants were instructed that they were free to allocate their study times and that they would be tested on a face recognition task after they had finished the study task. They were also encouraged by the experimenter to try their best to remember all the faces.

The self-paced face-learning task was controlled by the software E-Prime (version 1.1) and included three phases: self-paced study phase, distraction task, and face recognition phase. During the self-paced study phase, 20 faces (10 highly attractive and 10 less attractive faces) were presented sequentially in random order. Presentation of the final face was followed by a 2-min distraction task that consisted of solving simple arithmetic problems. In the face recognition phase, 40 faces (20 distracters and 20 targets) were displayed one at a time in random order. Participants were instructed to press two different keys (F/J) to indicate whether they had seen the presented face before [33]. The assignment of the keys to the two answers was counterbalanced across participants. After the experiment, we asked every participant two questions about the experiment (i.e., “Could you really imagine the scenario we provided? Could you guess the possible purpose of this study?”). All participants reported that they were able to imagine the scenario we provided. None of them guessed the real purpose of this study or the connection between the imagination and memory task. Finally, the participants were debriefed and dismissed.

### 2.2. Results

#### 2.2.1. Manipulation check

To evaluate the effectiveness of the manipulation, we compared the valence and arousal of emotion, romantic feelings, mating motivation, and sexual arousal between the two groups by independent-samples *t*-test [32]. As expected, participants in the mate-search condition reported greater sexual arousal (*M* = 4.27, *SE* = 0.28) than in the happiness-control condition (*M* = 2.43, *SE* = 0.31), *t*(27) = 4.39, *p* < 0.001; more romantic feelings (*M* = 4.00, *SE* = 0.34) than in the happiness-control condition (*M* = 2.86, *SE* = 0.35), *t*(27) = 2.36, *p* < 0.05; and greater motivation to seek a mate (*M* = 5.27, *SE* = 0.23) than in the happiness-control condition (*M* = 3.14, *SE* = 0.46), *t*(27) = 4.17, *p* < 0.005. No significant differences between conditions were found for valence (mate-search: *M* = 5.07, *SE* = 0.18; happiness-control: *M* = 5.36, *SE* = 0.23) and arousal of emotion (mate-search: *M* = 4.00, *SE* = 0.39; happiness control: *M* = 4.71, *SE* = 0.45), *p*s > 0.05.

#### 2.2.2. Recognition performance

The mean correct percentages for face recognition were calculated. A 2 (attractiveness: highly attractive vs. less attractive) × 2 (condition: mate-search vs. happiness-control) repeated-measures ANOVA revealed a significant effect of attractiveness, *F*(1,27) = 16.37, *p* < 0.001, *η* ^*2*^ = 0.38. In particular, the mean correct percentages for highly attractive faces (*M* = 0.84, *SE* = 0.02) reliably exceeded the mean percentages for less attractive faces (*M* = 0.77, *SE* = 0.02). There was neither a significant main effect of condition, *F*(1,27) = 0.38, *p* = 0.54, *η* ^*2*^ = 0.01, nor a significant interaction effect between attractiveness and condition, *F*(1,27) = 0.12, *p* = 0.73, *η* ^*2*^ = 0.004.

#### 2.2.3. Self-paced study times

**Fig 3:**
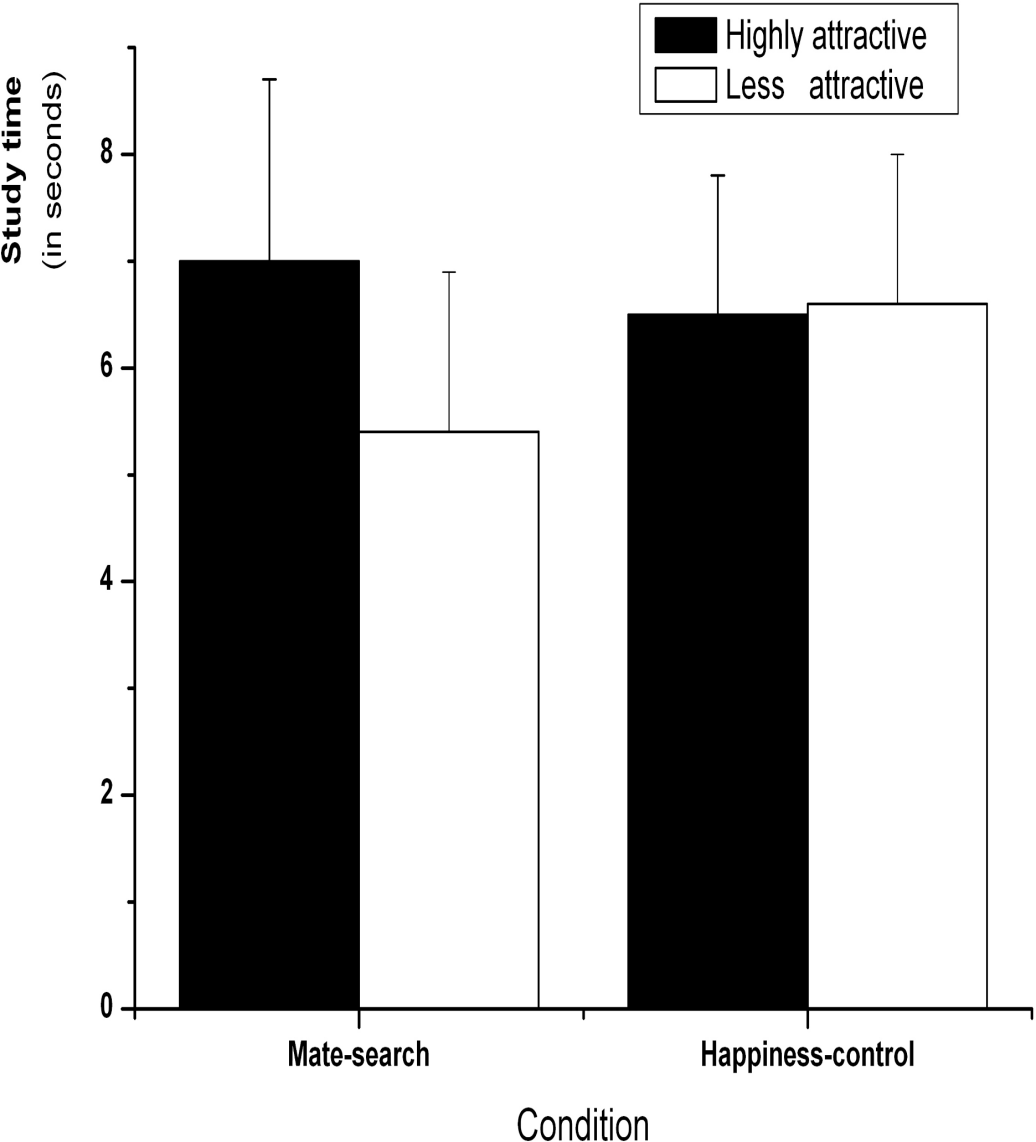
Mean study times (in seconds) allocated to highly and less attractive female faces for the mate-search and happiness-control conditions in Experiment 1. Error bars represent standard error of the mean.

The mean self-paced study times for highly attractive and less attractive faces were computed to examine whether the mate-search motives activation had an influence on the study-time allocation for the different sets of faces, as measured by the average study time (in seconds) for each face (Fig 3). A 2 (attractiveness: highly attractive vs. less attractive) × 2 (condition: mate-search vs. happiness-control) repeated-measures ANOVA revealed a significant main effect of attractiveness, *F*(1, 23) = 7.98, *p* < 0.05, *η* ^*2*^ = 0.26, showing that highly attractive faces (*M* = 6.77, *SE* = 1.09) were allocated more time than less attractive faces (*M* = 5.99, *SE* = 1.01). There was no significant main effect of condition, *F*(1,27) = 0.03, *p* = 0.85, *η* ^*2*^ = 0.01, but there was a significant interaction between attractiveness and condition, *F*(1,27) = 10.15, *p* < 0.005, *η* ^*2*^ = 0.27. A simple effect test revealed that participants allocated more time to study highly attractive (*M* = 6.98, *SE* = 1.74) than less attractive faces (*M* = 5.39, *SE* = 1.50) in the mate-search condition, *t*(14) = 3.77, *p* < 0.005, *d* = 0.24. However, the happiness-control condition yielded no significant differences in study time between highly attractive (*M* = 6.53, *SE* = 1.34) and less attractive faces (*M* = 6.65, *SE* = 1.37), *t*(13) = -0.36, *p* = 0.72.

### 2.3. Discussion

In accordance with our hypothesis, the participants whose mate-search motives were activated allocated more time to study highly attractive faces than less attractive faces, while participants in the control condition allocated equivalent time to the two groups of female faces. This result suggests that activating mate-search motives might influence individuals’ SRL processes.

These findings are consistent with an evolutionary psychological perspective about mating and adaptive behaviors [1–6]. The participants who imagined joining a dating party might have developed a specific bias to study highly attractive female faces rather than trying to remember less attractive faces. This study time allocation bias could indicate potential mating-related functions, that is, a greater chance to remember highly attractive female faces. On the other hand, because the happiness-control scenario was devoid of any romantic connotations and thus did not prime mate-related motives, no effects were observed for the control condition (also see another mini experiment in S1 File).

## Experiment 2

In Experiment 2, we sought to replicate the findings of Experiment 1 by activating female participants’ mate-guarding motives. Previous studies showed that same-sex individuals with highly attractive faces would be considered as more threatening rivals [4, 34–37]. Priming jealousy by an imagery task would activate the female participants’ mate-guarding motivation and induce adaptive cognitions or behaviors [4]. Therefore, similar to the influence of the mate-search motives in male participants, we predicted that activating the mate-guarding motives would lead the female participants to spend more time on studying highly attractive same-sex faces.

### 3.1. Methods

#### 3.1.1. Participants

Fifty undergraduate female students (mean age = 21.61 years, *SD* = 1.88 years) participated in Experiment 2. All participants were right-handed with normal vision. Participants were randomly assigned to the mate-guarding or anxiety-control group. All participants claimed that they were in a romantic relationship or had dated someone in the past [6].

#### 3.1.2. Materials

The female faces photographs used in this experiment were the same as in Experiment 1.

#### 3.1.3. Design and procedure

A 2 (attractiveness: highly attractive vs. less attractive) × 2 (condition: mate-guarding vs. anxiety-control) mixed design was adopted with condition as the between-subjects factor and attractiveness as the within-subject factor.

The procedure was the same as in Experiment 1, except for the guided imagery procedure. The participant in the mate-guarding condition was asked to visualize a friend telling her that her boyfriend was flirting with other females in a bar; if they were single, they were asked to imagine the same scenario with someone they had been dating in the past or currently had romantic feelings for [6]. For the anxiety-control condition, participants performed a similar task but the imagined boyfriend situation was replaced by an anxiety situation, in which they failed an important exam. After the imagery tasks, participants completed the manipulation check scales regarding their valence and arousal of emotion and jealousy level. Participants in two conditions were then introduced to the self-paced face-learning task with different introductions. For the mate-guarding condition, participants were requested to imagine that the face images belonged to 20 females who were in the same bar with their boyfriend, and one of them was flirting with their boyfriends. For the anxiety-control condition, participants were asked to imagine that the face images belonged to strangers.

After the experiment, we also asked every participant the same two questions as in Experiment 1. All participants reported that they were able to imagine the scenario we provided. None of them could guess the real purpose of this study or the connection between the imagination and memory task.

### 3.2. Results

#### 3.2.1. Manipulation check

To evaluate the effectiveness of the manipulation, we compared the valence and arousal of emotion and jealousy level between the two groups by an independent- samples *t*-test. As expected, participants in the mate-guarding condition reported a greater jealousy level (*M* = 4.44, *SE* = 0.34) than in the anxiety-control (*M* = 2.96, *SE* = 0.31), *t*(48) = 3.38, *p* < 0.001. No significant differences were found for valence (mate-guarding: *M* = 2.08, *SE* = 0.24; anxiety-control: *M* = 2.28, *SE* = 0.23) and arousal of emotion (mate-guarding: *M* = 2.80, *SE* = 0.35; anxiety-control: *M* = 2.96, *SE* = 0.36) between the two groups, *p*s > 0.05.

#### 3.2.2. Recognition performance

The mean correct percentages for face recognition were calculated. A 2 (attractiveness: highly attractive vs. less attractive) × 2 (condition: mate-guarding vs. anxiety-control) repeated-measures ANOVA revealed a significant main effect of attractiveness, *F*(1,48) = 36.64, *p* < 0.001, *η* ^*2*^ = 0.43. In particular, the mean correct recognition percentages for highly attractive faces (*M* = 0.90, *SE* = 0.01) significantly exceeded mean correct percentages for less attractive faces (*M* = 0.79, *SE* = 0.03). Neither asignificant main effect of condition, *F*(1,48) = 0.002, *p* = 0.97, *η* ^*2*^ < 0.001, nor a significant interaction effect between attractiveness and condition were found between the two groups, *F*(1,48) = 0.003, *p* = 0.96, *η* ^*2*^ < 0.001.

#### 3.2.3. Self-paced study times

**Fig 4:**
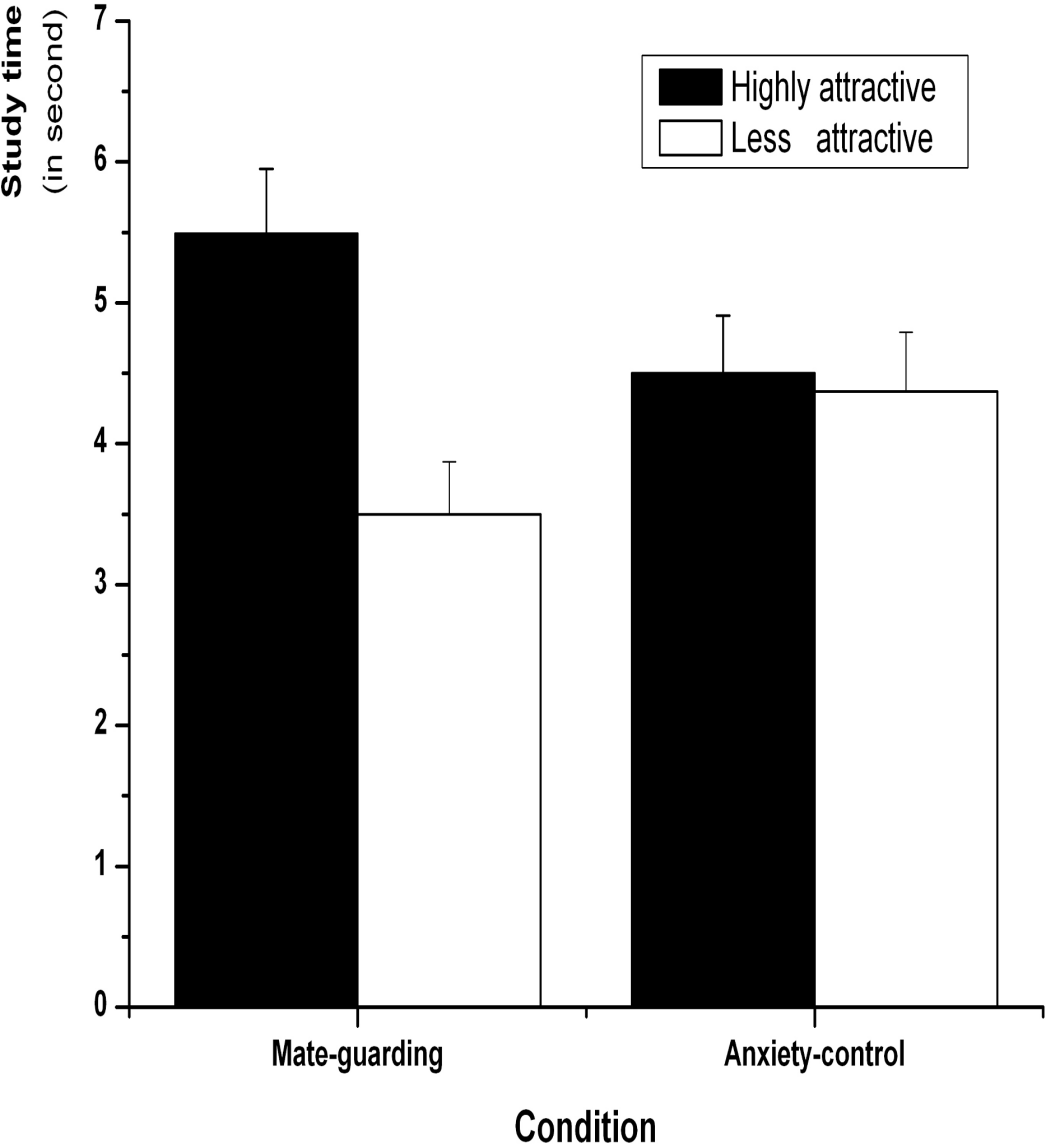
Mean study times (in seconds) allocated to highly and less attractive female faces for the mate-guarding and anxiety-control conditions in Experiment 2. Error bars represent standard error of the mean.

Mean self-paced study times for highly and less attractive faces were computed to examine whether the mate-guarding goal activation had an influence on the study-time allocation for the two sets of faces (Fig 4). A 2 (attractiveness: highly attractive vs. less attractive) × 2 (condition: mate-guarding vs. anxiety-control) repeated-measures ANOVA revealed a significant main effect of attractiveness, *F*(1,48) = 59.39, *p* < 0.001, *η* ^*2*^ = 0.53, showing that highly attractive faces (*M* = 5.01, *SE* = 0.32) were allocated more time than less attractive faces (*M* = 3.93, *SE* = 0.28). There was no significant main effect of condition, *F*(1,48) = 0.05, *p* = 0.83. There was a significant interaction between attractiveness and condition, *F*(1,48) = 42.42, *p* < 0.001, *η* ^*2*^ = 0.47. The subsequent simple effect test revealed that participants allocated more time to studying highly attractive faces (*M* = 5.57, *SE* = 0.47) than less attractive faces (*M* = 3.56, *SE* = 0.38) in the mate-guarding condition, *t*(24) = 8.41, *p* < 0.001, *d* = 0.86. However, the anxiety-control condition showed no significant differences between study time for highly attractive (*M* = 4.50, *SE* = 0.41) and less attractive faces (*M* = 4.38, *SE* = 0.42), *t*(24) = 0.81, *p* = 0.43.

### 3.3. Discussion

The findings of Experiment 2 extended those of Experiment 1 by activating another type of mate-related motives, that is, mate-guarding goals. The results confirmed our hypothesis that when the priming condition referred to mate guarding and evoked participants’ jealously, the highly attractive faces were studied longer than the less attractive faces. The participants in the control condition allocated equivalent time to the two groups of female faces. This finding was in line with previous research, which argued that the mate-guarding cues activated the mate-guarding motives and directed the adaptive behavior bias towards highly attractive same-sex faces [4]. Taken together, the findings from Experiments 1 and 2 are consistent with the hypothesis that mating-related motives lead people to allocate more study time to highly attractive female faces.

## General Discussion

The current study showed that participants (Experiment 1: males; Experiment 2: females) whose mate-related motives (Experiment 1: mate-search motives; Experiment 2: mate-guarding motives) were activated allocated more time to study highly attractive female faces than less attractive ones, suggesting that mate-related motives priming might have an influence on SRL. To our knowledge, this is the first study to explore the relationship between mate-related motives and SRL, which can widen our views on both evolutionary psychology and SRL.

Evolutionary psychologists have suggested that mate-relevant information is processed by a highly specialized, content-specific information-processing module that has evolved as a solution to recurring fitness-relevant problems [1, 38]. The different strategies to allocate time for the same face materials revealed different motivational states between the participants in the mate-related and control groups. As revealed by previous research, mate-related motives (i.e., mate-search and mate-guarding) interacted with goal-relevant stimuli to influence the individual’s cognitive processes, such as attention [4], basic social categories [5], and memory [9]. In the current study, participants might also have activated mate-related motives and thus allocated more study time to highly attractive female faces, which could enhance the chance to solve fitness-relevant problems. In the mate-search condition in Experiment 1, if a male spent more time memorizing females with highly attractive faces, he would have a better chance of remembering those faces later, which could theoretically help him to develop some targeted strategies to flirt with the ideal girl. In Experiment 2, because the highly attractive same-sex faces imply a threat from potential rivals, spending more time to memorize these faces could help the female to remember them and adopt further strategies to guard her mate (e.g., keeping an eye on the attractive rivals who might visit her mate’s Facebook page or preventing her mate from meeting the rivals) [4, 36]. Although participants in both the mate-search and mate-guarding groups showed similar patterns in study-time allocation for faces, the underlying motives and goals are quite different, which has specific implications for reproductive fitness. These results are in accordance with previous theories and a burgeoning body of research about domain-specific motivations and behaviors [1].

The main results also indicate that the learning situation and motivational state affect study-time allocation. Individuals adopted different study-time allocation strategies to maximize the likelihood of efficiently obtaining their goals [22, 24]. Based on the theories of evolutionary psychology that we stated above, the participants in the mate-related conditions regulated their study-time allocation to maximize the chance to remember the highly attractive female faces. It is worth mentioning that, in our two experiments, although all participants were instructed to set up a learning goal to memorize all female faces, the participants whose mate-related goals were activated did not seem to follow the instruction, confirming the powerful influence of mate-related motivation [2].

An alternative explanation of these effects could involve the attentional process. Previous research revealed that mate-related motives induced the individuals’ attention bias toward highly attractive faces [4]. Having limited cognitive resources, the attentional focus will help the individuals to spend their limited resources to process the most relevant information. However, in our view, the attentional process cannot solely explain the present results. As stated in the introduction section, SRL represents learning that is guided by metacognition, strategic action, and motivation to learn. Reflected in the self-paced study process, SRL refers to learners’ decision about when to begin and terminate the study process and how long to spend on specific items, which involves many other cognitive and behavioral components (e.g., monitoring, choice, decision-making). According to the agenda-based regulation model, which was recently proposed by Ariel et al. to explain SRL [22], learners’ study decisions were guided by an agenda that they developed to prioritize and allocate time for items to study. Indeed, attention plays an important role in the SRL process, because it helps individuals to maintain their learning agenda during the self-paced study process. However, the SRL process does not merely involve attentional processes. As for the experimental conditions in the current study, the participants in the mate-related conditions might have constructed an agenda (either consciously or unconsciously) to remember the highly attractive female faces. When they performed the self-paced study task, the participants might have allocated more attention to highly attractive faces and decided to spend more time remembering them, because this learning strategy was in accordance with the agenda. When they attended to the less attractive faces, they likely terminated their study earlier, because remembering less attractive faces would not help the attainment of the learning goal (i.e., remembering more attractive female faces). Therefore, the attention process plays an important role in the SRL process, just like the other processes (e.g., memory, decision making). Further research should try to clarify the role of attentional processes by analyzing eye-tracking and behavioral data under similar conditions as in the current study.

Another interesting finding of this study was the improved memory for the highly attractive faces regardless of the context that was primed in both experiments. That is, facial attractiveness had a different effect on the two dependent variables, namely memory performance and study-time allocation. On one hand, the finding of improved memory for highly attractive faces agrees with previous research [39]; for example, Marzi and Viggiano adopted a face-recognition task and found that highly attractive faces were remembered better than less attractive faces [39]. On the other hand, we found that participants spent more time to study highly attractive faces in the mating conditions, but did not show better recognition of these faces. It seems that even though more study time was allocated, it did not aid in recognition. This finding might reflect the so-called “labor-in-vain effect” (i.e., increased study-time on specific items yields little or no improvement in memory performance) [40–42], that was first described by Nelson and Leonesio [40]. In their three experiments, the participants in the accuracy-emphasis group spent more time (ranging from twice as long in Experiment 1 to seven times as long in Experiment 3) to study items than those in the speed-emphasis group. However, the two groups showed little or no reliable difference in recall performance. This effect was confirmed in subsequent research using different learning materials and under different task constraints [41–43]. For instance, Mazzoni and Cornoldi examined whether they could eliminate the labor-in-vain effect. Their findings showed that the strategy of time allocation was influenced by manipulations of the task constriaints. However, none of these manipulations eliminated the labor-in-vain effect [41]. Metcalfe argued that there are two necessary conditions for the control of study to be helpful: monitoring must be accurate and appropriate choices must be implemented during study [21]. Considering that an individual’s metacognitive monitoring might sometimes not be accurate, one of the possible reasons for a labor-in-vain effect could be learners’ inadequate monitoring during self-paced study [30]. Another explanation for this effect might be the “smoke detector principle”. This states that, where the potential payoff of a correct detection greatly outweighs the cost of a false alarm, the sensitivity of the mechanism should be high [44]. In the current study, the reproductive fitness problem is so vital that individuals are predicted to choose a strategy that risks wasting effort and time on false alarms because the payoff of a correct detection of sexual opportunity or a mate guarding situation from an attractive woman are very high.

The current study highlights the integration of social cognition and SRL. Most of the materials used in traditional SRL (metacognition) studies were noun-noun pairs or single words [22]. The findings based on these materials are difficult to generalize to tasks involving learning items outside of the semantic realm [40]. Some researchers acknowledge that we need to extend the research field of SRL to the social realm [45, 46]. However, this line of research has not been fully incorporated into the SRL (or metacognition) tradition. The current research confirms the hypothesis that activating mate-related goals had an influence on SRL and provides additional empirical evidence. Further studies could differentiate the influence of various social cognition components on SRL.

Another important implication of this study is that the findings may shed light on the unconscious process of SRL. Although self-regulated learning has been widely investigated ever since it was introduced in the 1980s [11], the degree of consciousness of this process has not received much attention. However, Ariel and Dunlosky found that learners’ study decisions could be influenced by two kinds of processing: agenda-based process (which was thought to be voluntarily controlled and conscious) and habitual process (which was thought to be automatic and unconscious), suggesting that self-regulated learning might occur either consciously or unconsciously [47]. In the current study, we found that the priming effect of mate-related motives on study-time allocation was significant and none of the participants realized the real connection between the imagination and face-study task, or the real intention of this study. As Reder et al argued, it is important to distinguish between the strategy-selection processes (study-time allocation) and the strategy itself [48]. We argue that participants in the mating-related groups might have been unaware about what caused them to allocate more time to the highly attractive faces. Although we make no claim that the participants in this study were unaware about the study strategies they adopted, our results together with previous studies suggest that there might be an unconscious process that affects self-regulated learning.

There are several limitations of this study. First, we did not examine gender differences in the two experiments, which might limit the generalizability of the findings [6, 49]. Based on the theories of sexual selection and investment, a highly attractive opposite-sex face is an important index for the male (but not for the female) intending to find a mate. The present study indicated that the males in the mate-search group spent more time on attractive female faces, which supports the sexual selection theory [6]. Future research could benefit from specifically testing gender differences in the two experiments by using male and female face photographs. Second, we did not ascertain possible interactions of mate-related motives priming and individual differences such as sociosexual orientation [6, 50]. For instance, in a recent study, Ritter et al. found that self-regulation capacity (ego depletion) plays an important role in romantically involved individuals’ attention process toward attractive faces [51]. Further research will be necessary to better understand the effects of individual differences on study-time allocation. Additionally, although all participants in the mate-guarding group claimed to have successfully envisioned the scenario, it is unclear whether additional factors, such as degree of relationship satisfaction and jealousy level, had moderating effects on the results. Furthermore, to make the anxiety condition in Experiment 2 more congruent with the mate-guarding condition, further research could add the instruction that the faces belonged to women who did better in the exam than the participant to the current imagery instruction.

In summary, the current study provides evidence that mate-related motives affect study-time allocation to female faces varying in attractiveness. In our opinion, the present research illustrates that bridging the perspectives of evolutionary psychology and SRL may deepen the understanding of both fields.

## Supporting Information

S1 Fig Mean study times (in seconds) allocated to highly and less attractive female faces for the three conditions in mini experiment. Error bars represent standard error of the mean.
(TIF)

S1 File Mini experiment for Experiment 1. (DOC)
