# Supplementary material for: Out of Lust or Jealousy: The Effects of Mate-Related Motives on Study-Time Allocation to Faces Varying in Attractiveness
Source: PLoS One. 2015 Jun 29;10(6):e0132207. doi: 10.1371/journal.pone.0132207 (PMC4485464; doi:10.1371/journal.pone.0132207)
Supplement: S1 File — (DOC) [file pone.0132207.s002.doc]

**Mini experiment for Experiment 1**

**1. Introduction**

In the current study, we followed the principle of mate-related motive priming procedure. That is, the mate-search condition in Experiment 1 contained mate-related cues (i.e., dating party); on the contrary, the happiness-control condition was devoid of any romantic connotations and mating cues [1-2]. Therefore, the effects could only explain by the mating-related motives only. However, as the reviewer suggested, that the scenarios (mate-search and happiness-control) where participants would meet these girls were quite different, which may have affected the way they remembered the faces in the following face study task. we designed a new-control condition in Experiment 1 to clarify the effects.

In this mini experiment, we request the male participants imagine a scenario where they would *meet all the women at the same time* (similar to the pattern in the mate-search groups), but this control scenario was devoid of any romantic connotation or mating cue. We guided the male participants to imagine getting a part-time job as a tour guide in their city, and having a pleasant day with a group of tourists with married couples consisting of pregnant women and their husbands. Pregnant women should be less attractive, since they are infertile while pregnant (therefore this scenario should not activate mate-search motives) [3]. The participants had to remember these women’s photographs, and study-time allocation was recorded just as for the mate-search group. If the results were affected by the way the participants meet the women in the imagined scenario (one by one, or all at the same time), there should be an interaction effect between condition (happiness-control vs. new-control condition) and face attractiveness on study-time allocation. In addition, the interaction effect between the mate-search and new-control group on study-time allocation should be insignificant.

**2. Participant**

Ten Chinese undergraduate students (mean age=23.2 years, *SD*=1.93 years) were recruited to participate in this new control experiment.

**3. Results**

***Manipulation check***

To evaluate the effectiveness of the manipulation, we compared the valence and arousal of emotion, romantic feelings, mating motivation, and sexual arousal between the three groups (mating-search vs. happiness-control vs. new-control) by a one-way ANOVA. As expected, participants in the mate-search condition reported greater sexual arousal, romantic feelings, and motivation to seek a mate than those in the control conditions. No significant differences were found for sexual arousal or romantic feelings between the happiness-control and new-control condition (*p*s>0.05).

No significant differences were found for valence and arousal of emotion between the three groups, *p*s > 0.05.

***Self-paced study times***

Mean self-paced study times for highly and less attractive faces were computed to examine whether the mate-search goal activation had an influence on the study-time allocation for highly attractive faces (measured by average study time spent on each face type, in seconds). A 2 (face-attractiveness: highly attractive vs. less attractive) × 3 (condition: mate-search vs. happiness-control vs. new-control) repeated-measures ANOVA revealed a significant interaction (S1 Fig), *F*(1,36) = 4.96, *p* < 0.01, *η^2^* = 0.22.

As the primary goal of this new experiment was to explore the potential effect of different ways to meet the women in the imagined scenario, a 2 (face-attractiveness: highly attractive vs. less attractive) × 2 (condition: mate-search vs. new-control) repeated-measures ANOVA revealed a significant interaction, *F*(1,23) = 5.84, *p* < 0.05, *η^2^* = 0.23. A 2 (face-attractiveness: highly attractive vs. less attractive) × 2 (condition: happiness-control vs. new-control) repeated-measures ANOVA was adopted, which resulted in an insignificant interaction between the two factors, F(1,22) = 0.16, p > 0.05.

**4. Discussion**

The results were in accordance with our hypothesis. Based on the ANOVA and the pattern revealed in S1 Fig, the main results indicate that study-time allocation was not affected by the way to meet the women in the imagined scenario.

Although the new-control scenario provided a chance for participants to “meet” all the women at the same time, for most male participants, the interest to finding a potential mate among pregnant women with their husband is low and carries some risk [3]. Therefore, the reported effects can be explained by the mating-related motives and the adaptive processing of specific information (highly attractive female faces).

The participants in the new-control condition seemed to spend less study time than the original two groups. The group differences did not reach significance level, and we speculate that this phenomenon could be attributed to the participants being recruited from different departments.

**References**

1. Maner JK, Gailliot MT, Rouby DA, Miller SL. Can't take my eyes off you: Attentional adhesion to mates and rivals. J Pers Soc Psychol. 2007; 93: 389–401.

2. Sundie JM, Kenrick DT, Griskevicius V, Tybur JM, Vohs KD, Beal DJ. Peacocks, Porsches, and Thorstein Veblen: Conspicuous consumption as a sexual signaling system. J Pers Soc Psychol. 2011; 100: 664-680.

3. Marlowe F, Wetsman A. Preferred waist-to-hip ratio and ecology. Pers Individ Dif. 2001; 30: 481-489.
